# Supplementary material for: Influence of Parturition on Rumen Bacteria and SCFAs in Holstein Cows Based on 16S rRNA Sequencing and Targeted Metabolomics
Source: Animals (Basel). 2023 Feb 21;13(5):782. doi: 10.3390/ani13050782 (PMC10000066; doi:10.3390/ani13050782)
Supplement: Supplementary file 1 [file animals-13-00782-s001.zip › Supplemental Figure S1.pdf]

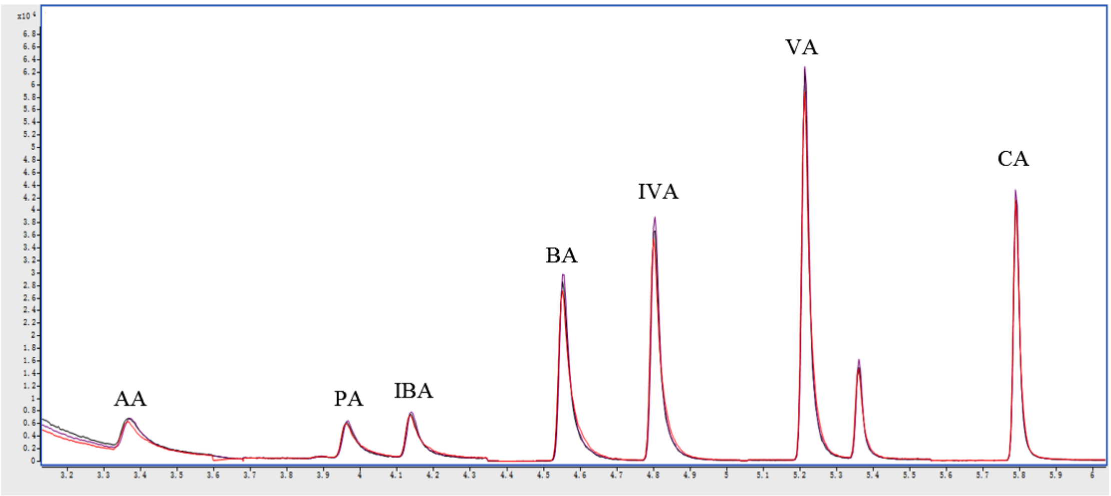

Figure S1. Total ion current (TIC) overlap of quality control sample (QC) mass spectrometry.

Note: The x-axis is the retention time of short-chain fatty acids (SCFAs), and the y-axis is the ion current intensity. The peak area of each chromatographic peak represents the relative content of the corresponding SCFA. AA: acetic acid; PA: propionic acid; IBA: isobutyric acid; BA: butyric acid; IVA: isovaleric acid; VA: valeric acid; Caproic acid (CA).
