# Supplementary material for: Influence of Parturition on Rumen Bacteria and SCFAs in Holstein Cows Based on 16S rRNA Sequencing and Targeted Metabolomics
Source: Animals (Basel). 2023 Feb 21;13(5):782. doi: 10.3390/ani13050782 (PMC10000066; doi:10.3390/ani13050782)
Supplement: Supplementary file 1 [file animals-13-00782-s001.zip › Supplemental Table S1.pdf]

**Supplemental Table S1.** The TMR diet formula of dairy cows before parturition

| Composition | Content (%) | Item | Unit (%) |
|---|---|---|---|
| Whole corn silage | 32.01 | Crude Protein | 14.51 |
| Soybean hulls | 12.46 | Starch | 17.96 |
| Alfalfa hay | 5.88 | NDF | 39.01 |
| Rice straw | 8.51 | Fat | 3.81 |
| Soybean meal-protein | 9.02 | NEL (MJ/kg) | 5.78 |
| Oat grass hay | 12.54 | | |
| Premix[1] | 4.25 | | |
| Cottonseed-whole | 6.95 | | |
| Corn-fine | 1.80 | | |
| Calcium carbonate | 0.23 | | |
| Magnesium sulfate | 0.35 | | |
| Sugar cane | 1.97 | | |
| Methionine | 0.19 | | |
| Puffed soy flour | 2.86 | | |
| Yeast polysaccharide | 0.98 | | |

[1]Per kilogram of premix contains the following: VA 325 KIU, VE 2000 mg, VD 95 KIU, Co 18 mg, Se 10 mg, I 30 mg, Mn 1600 mg, Zn 800 mg, Cu 300 mg, Fe 400 mg, Ca 18.0%, P 6.8%.
