# Supplementary material for: Influence of Parturition on Rumen Bacteria and SCFAs in Holstein Cows Based on 16S rRNA Sequencing and Targeted Metabolomics
Source: Animals (Basel). 2023 Feb 21;13(5):782. doi: 10.3390/ani13050782 (PMC10000066; doi:10.3390/ani13050782)
Supplement: Supplementary file 1 [file animals-13-00782-s001.zip › Supplemental Table S2.pdf]

Supplemental Table S2. GC-MS/MS testing conditions for short chain fatty acids (SCFAs) in rumen fluid sample

Items	Parameter
Injection volume	2 μL
Front Inlet Mode	Splitless
Carrier Gas	Helium
Column	DB-FFAP (30 m × 0.25 mm × 0.25 μm)
Column Flow	1.2 min^{-1}
Oven Temperature Ramp	Holds on 1 min at 95 °C, raises to 100 °C at a rate of 25 °C/min, raises to 130 °C at a rate of 17 °C/min, holds on 0.4 min, raises to 200 °C at a rate of 25 °C/min, holds on 0.5 min, running for 3 min.
Front Injection Temperature	200 °C
Transfer Line Temperature	230 °C
Ion Source Temperature	230 °C
Quad Temperature	150 °C
Electron Energy	70 eV
Scan mode	MRM
Solvent Delay	3.0 min
