# Supplementary material for: Influence of Parturition on Rumen Bacteria and SCFAs in Holstein Cows Based on 16S rRNA Sequencing and Targeted Metabolomics
Source: Animals (Basel). 2023 Feb 21;13(5):782. doi: 10.3390/ani13050782 (PMC10000066; doi:10.3390/ani13050782)
Supplement: Supplementary file 1 [file animals-13-00782-s001.zip › Supplemental Table S3.pdf]

Supplemental Table S3. Precision and recovery rate of QC (quality control) samples

Component	Intra-day precision RSD%		Inter-day precision RSD%		Recovery %		
	LQC	HQC	LQC	HQC	LQC	MQC	HQC
Acetic acid (AA)	2.29	2.1	3.4	1.58	104.63	93.58	92.23
Propionic acid (PA)	0.55	1.82	1.57	1.45	105.09	102.3	102.66
Isobutyric acid (IBA)	1.28	1.34	1.26	3.07	99.98	87.42	109.3
Butyric acid (BA)	1.37	1.25	1.86	2.99	93	101.49	109.72
Isovaleric acid (IVA)	0.42	1.94	1.75	3.25	94.4	109.28	108.36
Valeric acid (VA)	1.33	3.64	1.2	3.36	110.21	108.81	110.67
Caproic acid (HA)	0.5	2.29	1.12	2.49	99.51	106.6	109.99

Note: LQC, MQC and HQC respectively mean low, middle and high concentrations of QC samples.
