# Supplementary material for: Influence of Parturition on Rumen Bacteria and SCFAs in Holstein Cows Based on 16S rRNA Sequencing and Targeted Metabolomics
Source: Animals (Basel). 2023 Feb 21;13(5):782. doi: 10.3390/ani13050782 (PMC10000066; doi:10.3390/ani13050782)
Supplement: Supplementary file 1 [file animals-13-00782-s001.zip › Supplemental table S4.pdf]

Supplemental Table S3. The concentrations of each SCFA in each rumen fluid sample (μg/mL)

Sample	AA	BA	CA	IBA	IVA	PA	VA
E1	1170	569	51	47.4	43.9	661	88.8
E2	874	467	24.1	35.3	36	631	61.8
E3	775	440	23.1	30.8	30.2	490	59.7
E4	839	432	21.9	21.9	24.5	452	49.3
E5	1000	482	37.5	38.4	32.7	671	73.4
E6	1020	506	25.7	45.6	37.3	664	76.5
E7	941	486	28.5	32.3	26.1	639	65.6
E8	846	534	35.8	34.3	27.6	606	72.1
E9	1080	648	35.5	19.1	41.3	633	81.3
E10	1030	654	27.7	34.3	45.9	662	88.2
A1	687	324	9.26	40.8	39.6	335	43.8
A2	1080	478	12.4	82.9	77.9	571	68
A3	561	271	12	29	25.5	265	31.4
A4	958	464	21.6	56.1	47	481	64.1
A5	674	366	13.5	32.6	30.9	344	40.4
A6	248	133	4.77	11	12.2	108	12
A7	149	81.7	2.57	7.39	8.7	70.8	10.1
A8	950	597	35.5	64	59.8	558	79.1
A9	373	258	16.4	16.2	22.4	186	33.5
A10	660	309	13.7	26.4	25.6	318	34.4

Note: AA: acetic acid; PA: propionic acid; IBA: isobutyric acid; BA: butyric acid; IVA: isovaleric acid; VA:valeric acid; CA: caproic acid.
