# Supplementary material for: Influence of Parturition on Rumen Bacteria and SCFAs in Holstein Cows Based on 16S rRNA Sequencing and Targeted Metabolomics
Source: Animals (Basel). 2023 Feb 21;13(5):782. doi: 10.3390/ani13050782 (PMC10000066; doi:10.3390/ani13050782)
Supplement: Supplementary file 1 [file animals-13-00782-s001.zip › Supplemental Table S5.pdf]

Supplemental Table S4. Tags and average length of rumen bacteria communities

Sample	Raw Tags	Clean Tags	Effective Tags	Avglen (nt)	Effective (%)
E.1	72981	70250	45576	411	62.45
E.2	99283	81838	60907	415	61.35
E.3	78549	75283	44790	416	57.02
E.4	71543	69008	43263	413	60.47
E.5	73507	69998	43119	410	58.66
E.6	67065	64420	39953	416	59.57
E.7	78668	74043	48385	416	61.51
E.8	61660	59166	34587	415	56.09
E.9	66149	63872	42892	413	64.84
E.10	70234	67902	42512	412	60.53
A.1	61033	58173	36660	410	60.07
A.2	63719	61306	38665	413	60.68
A.3	60766	56768	35336	414	58.15
A.4	74278	70419	42469	416	57.18
A.5	62060	59725	39544	411	63.72
A.6	72758	69166	41202	415	56.63
A.7	70350	65965	42287	412	60.11
A.8	58820	56637	34908	412	59.35
A.9	56811	53679	35156	412	61.88
A.10	79198	70832	45668	411	57.66
Total	1399432	1318450	837879	413	59.90
